# Agency over a phantom limb and electromyographic activity on the stump depend on visuomotor synchrony: a case study

**DOI:** 10.3389/fnhum.2014.00545

**Published:** 2014-07-29

**Authors:** Shu Imaizumi, Tomohisa Asai, Noriaki Kanayama, Mitsuru Kawamura, Shinichi Koyama

**Affiliations:** ^1^Graduate School of Engineering, Chiba UniversityChiba, Japan; ^2^Japan Society for the Promotion of ScienceTokyo, Japan; ^3^NTT Communication Science Laboratories, NTT CorporationKanagawa, Japan; ^4^Institute of Biomedical and Health Sciences, Hiroshima UniversityHiroshima, Japan; ^5^School of Medicine, Showa UniversityTokyo, Japan; ^6^Division of Systems and Engineering Management, Nanyang Technological UniversitySingapore

**Keywords:** phantom limb, motor sensation, sense of agency, delayed visual feedback, mirror therapy

## Abstract

Most patients, post-amputation, report the experience of a phantom limb. Some even sense voluntary movements when viewing a mirror image of the intact limb superimposed onto the phantom limb. While delayed visual feedback of an action is known to reduce a sense of agency, the effect of delayed visual feedback on phantom motor sensation (i.e., sense of controlling a phantom limb) has not been examined. Using a video-projection system, we examined the effect of delayed visual feedback on phantom motor sensation in an upper-limb amputee (male; left upper-limb amputation). He was instructed to view mirrored video images of his intact hand clasping and unclasping during a phantom limb movement. He then rated the intensity of the phantom motor sensation. Three types of hand movement images were presented as follows: synchronous, asynchronous with a 250-ms delay, and asynchronous with a 500-ms delay. Results showed that phantom motor sensation decreased when the image was delayed by 250 and 500 ms. However, when we instructed the patient to adjust the phase of phantom limb movement to that of the image with a 500-ms delay, phantom motor sensation increased. There was also a positive correlation between intensity of phantom motor sensation and electromyographic (EMG) activity on deltoids at the patient’s stump. These results suggest that phantom motor sensation and EMG activity on the stump depend on visuomotor synchrony and top-down effects.

## Introduction

We may often feel that an action triggers a change in the external environment. Such feelings are referred to as a “sense of agency” (Gallagher, 2000; David et al., 2008; Moore and Obhi, 2012). Although recent studies have described many aspects to a sense of agency (e.g., Synofzik et al., 2008), the key concept is a sense of controlling one’s own actions and, therefore, the external proxy or tool (Haggard and Chambon, 2012). A sense of agency emerges not only through voluntarily generating an action but also by having temporal contiguity between the action and outcome (Blakemore et al., 1999; Bays et al., 2005). The internal forward model in the central motor system has been adopted to help explain the origin of this sense of agency based on sensorimotor processes (Wolpert et al., 1995; Wolpert, 1997; Frith et al., 2000). This model is based on efference copy, which is generated as a copy of the motor commands from a self-produced action (von Holst and Mittelstaedt, 1950) and predicted sensory consequences of motor commands before actual afferent feedback. These consequences are matched against actual feedback. If this prediction does not match the feedback, the perceived sense of agency will decrease. For instance, previous studies suggest that delayed visual feedback when voluntarily handling tools decreases a sense of agency (Franck et al., 2001; Asai and Tanno, 2007).

Although there are several studies assessing a sense of agency manifesting with *intact* limbs, few studies have approached how a sense of agency emerges with *affected* limbs (i.e., phantom limbs). After the amputation of a limb, up to 98% of patients report phantom limb awareness (Ramachandran and Hirstein, 1998). Furthermore, 50–80% of amputees feel pain at the phantom limb site (Kooijman et al., 2000). It has been suggested that neural plasticity plays a key role in the emergence of phantom limb sensations (Flor et al., 2006, 2013). Some patients report that they can voluntarily move the phantom limb. Voluntary movements of a phantom limb have been described as imaginary movements (Ersland et al., 1996; MacIver et al., 2008). Recently, Raffin et al. (2012a) demonstrated that amputees are capable of performing motor execution and motor imagery with their phantom limbs. Furthermore, they suggest that distinct cortical networks contribute to voluntary phantom motor execution (Raffin et al., 2012b). However, it is still unclear how an individual produces voluntary movement in a phantom limb.

Voluntary movement of a phantom limb could be interpreted based on a sense of agency. Ramachandran and Rogers-Ramachandran (1996) developed a technique for providing visual feedback of a moving, intact limb corresponding to the phantom limb while using a mirror placed upright on a table and vertical to an amputee’s chest (mirror therapy). The amputee moves his/her intact limb and looks at the mirrored image of the intact limb superimposed on the phantom limb. Subsequently, the amputee can experience control of the phantom limb. Blakemore et al. (2002) suggested that mirrored visual feedback matches sensory feedback predicted by the forward model. Coincidently, this is an efference copy of motor commands generating change in the predicted position of the phantom limb corresponding to mirrored visual feedback.

Mirror therapy was originally developed to relieve phantom limb pain (Ramachandran and Rogers-Ramachandran, 1996; Chan et al., 2007). During therapy, amputees view their unaffected limb in a mirror so that the mirrored image superimposes on their contralateral affected limb behind the mirror. Amputees are encouraged to synchronize their phantom limb with visual feedback from the mirror. Visual feedback of a mirrored-intact limb provides predicted sensory feedback corresponding to motor commands; consequently, this coherent sensorimotor integration might alleviate phantom limb pain (Harris, 1999; McCabe et al., 2005; Ramachandran and Altschuler, 2009). Because a sense of agency plays an important role in the formation of a coherent body image based on visuo-proprioceptive interaction (Tsakiris et al., 2006), it is assumed that a sense of agency is an important factor for determining the effects of mirror therapy and an individual’s ability to move a phantom limb. However, few studies have directly examined the relationship between sense of agency and phantom limb movements.

A previous study suggested that phantom limb pain was attenuated when amputees perceived sense of agency toward a moving hand-image superimposed on a phantom limb in an immersive virtual environment (Cole et al., 2009). Conversely, phantom limb pain did not decrease when amputees did not perceive a sense of agency. While Cole et al. (2009) suggest that a sense of agency is linked to alleviating phantom limb pain, the authors used semi-structured interviews to assess (rather than quantify) a sense of agency. Moreover, the authors had no means to modulate an induced sense of agency. To manipulate and investigate a sense of agency, previous studies adopted systematically delayed visual feedback of an action from an intact limb (Franck et al., 2001; Asai and Tanno, 2007). For instance, participants were asked to judge whether they felt they had produced the action outcome. The proportion of “yes” responses was an index of a sense of agency. The authors then quantitatively showed that longer visual feedback delays decreased the sense of agency. Other studies have measured a sense of agency by asking participants to rate, on a numerical scale, to what extent the participant felt that he/she was the one who caused the action outcome (e.g., Sato and Yasuda, 2005).

The present case study examined how temporal congruence between phantom limb movement and visual feedback modulates a sense of controlling a phantom limb (i.e., a sense of agency over a phantom limb). We refer to this sensation as “phantom motor sensation” in order for the amputee to quantify sensory information more easily. Our first hypothesis was that the delayed visual feedback on phantom movement decreases or even extinguishes phantom motor sensation. We also examined how phantom motor sensations could be modulated by spontaneous effort to adjust phantom limb movement to delayed visual feedback. Previous studies suggested that a sense of effort could facilitate a sense of agency among individuals with intact limbs (Demanet et al., 2013; Damen et al., 2014). When an individual’s action can be seen as his/her own action, he/she can misattribute the viewed action, which can affect actual movement (Nielsen, 1963; Fourneret and Jeannerod, 1998). Thus, our second hypothesis was that a reduction in the subjective intensity of phantom motor sensation by delayed visual feedback would be less when an amputee receives an instruction to adjust the phantom limb movement phase to that of the delayed feedback. Finally, our third hypothesis was that phantom motor sensations positively correlate with electromyographic (EMG) activity on stump muscles. Previous studies found that stump muscles are activated during phantom motor execution, and waveforms show consistent phases with that of phantom limb movement (Reilly et al., 2006; Gagné et al., 2009; Kawashima and Mita, 2009; Raffin et al., 2012a; Kawashima et al., 2013). Furthermore, muscles are inactive during motor imagery (Raffin et al., 2012a). A recent study also suggested that subjective movability of a phantom limb is positively correlated with EMG activity in stump muscles (Kawashima et al., 2013).

To test our hypotheses, we investigated an upper-limb amputee who obtained visual feedback from filmed images of intact limb movements. This is a technique used in previous studies (Giraux and Sirigu, 2003; Mercier and Sirigu, 2009) that has been validated as an alternative to traditional mirror therapy. This technique allowed the manipulation of the presentation onset of visual feedback in milliseconds, as well as present delayed visual feedback of an action to the participant. To test the first and second hypotheses, subjective intensities of phantom motor sensation from the amputee were compared across conditions varying in visual feedback delay and the amputee’s mental set. Then, to test the third hypothesis, we analyzed the correlation between the intensities of phantom motor sensation and EMG activity on stump muscles. Finally, we confirmed that EMG responses from a group of control participants’ deltoids were not evoked by motor imagery (Gandevia et al., 1997; Hashimoto and Rothwell, 1999; Raffin et al., 2012a) with visual feedback.

## Materials and methods

### Case history

The patient was a 67-year-old right-handed male. He was recruited from the outpatient clinic of Showa University Hospital. His left arm was amputated 5 cm below the shoulder due to a car accident 39 years prior. He had been using a prosthesis for esthetic purposes. For 31 years after the accident, he had phantom limb pain with numbness and perceived his hand with a clenching spasm as frequently as 10 times a month. The pain lasted for a few hours on average and was very strong; once this pain arose, he was unable to work. Seven years before, he started mirror therapy for approximately 10 min per day, which relieved his pain. More recently, he experienced mild phantom limb pain about once a month, but pain was relieved by mirror therapy. After recovery from the phantom limb pain, he still had phantom limb experiences. Work with the patient for the current study was undertaken while he was not experiencing phantom limb pain. He had corrected-to-normal eyesight and no history of neurological or psychiatric illness except for the limb amputation and phantom limb sensations.

### Control participants

Six healthy male volunteers (age 22.33 ± 0.82 years) participated in the same EMG recording scenario as the patient. Five were self-declared right-handed, and one was left-handed. All had normal or corrected-to-normal eyesight and no history of neurological or psychiatric illness. Since it has been established that delayed visual feedback decreases a perceived sense of agency over intact limbs (e.g., Asai and Tanno, 2007), these volunteers participated only in the EMG recording portion of the experiment.

### Ethics statement

The ethics committee of the School of Medicine, Showa University, approved this study, which was conducted according to principles outlined by the Declaration of Helsinki. The patient and controls received an explanation of the research protocol and gave written informed consent.

### Experimental setup

**Figure 1:**
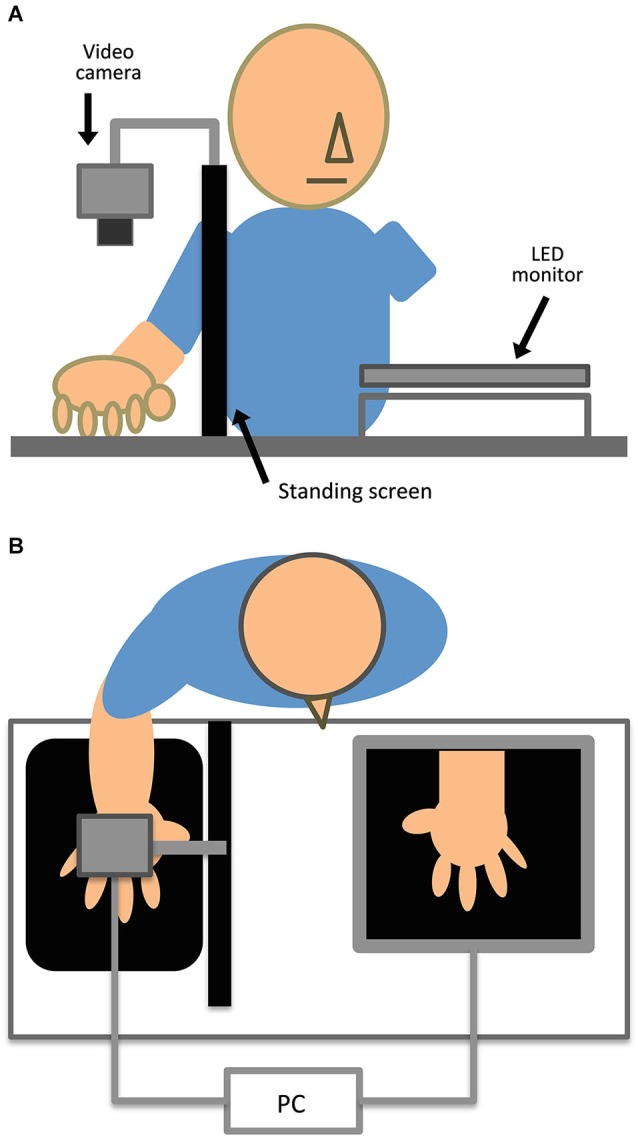
**Experimental setup depicted in (A) elevated and (B) horizontal views.** Participants sat at the table and placed their right hand on the tabletop. Control participants placed their left hand in the gap between the LED monitor and table. A steel framework fixed the video camera and black standing screen. LED lighting lit the space near the right hand.

This is shown in Figures 1A,B. A participant sat at a table and put his right elbow on the table. The dorsum of the right hand was recorded by a color video camera (STC-TC33USB-AS, Sensor Technologies America, Inc.) at 60 frames per second, from approximately 45 cm directly above the hand. The tabletop was covered with a black cloth so that the camera captured the hand isolated against a black background. To prevent the camera from capturing fluorescent light flickers in the room, LED lighting (Z-6600, Yamada Shomei Lighting, Ltd.) illuminated the space near the right hand to be captured.

We developed an image-processing program to generate video images with or without systematic delay (0, 250, or 500 ms) using Hot Soup Processor version 3.32 (Onion Software). These images were horizontally flipped so that the image of the right hand appeared to look like the left hand. The filmed images were processed, and then simultaneously displayed on a 23-inch LED monitor (i2353Ph, AOC) with a resolution of 1920 × 1080 pixels and a refresh rate of 60 Hz. The monitor was placed on a table in front of the participant with an 8 cm gap from the table surface. A personal computer (CF-SX1, Panasonic Corporation) controlled image recording with the camera, image processing, and stimulus presentation on the monitor. The hand images were presented at an almost identical size on the monitor. In the experiment with the control group, participants put their intact left hand into this gap. Participants sat with their head approximately 35 cm from the monitor. To obstruct the direct view of the right hand, each participant’s right hand was placed behind a black standing screen aligned with the mid-sagittal plane on the table.

### Procedure

We conducted tasks in which participants repetitively clasped and unclasped their right hand for 10 s set to a metronome (120 beats per minute). At the same time, the patient intended to move his phantom limb at the same rate as the right hand. Control participants were to *imagine* the movement of their left hand in the same manner. The experimenter told the participants to start and stop the task. The reason for using the clasp/unclasping task was that the patient was familiar with this task for his mirror therapy.

The study consisted of five experimental and two control conditions. The patient performed a single trial in each experimental and control condition, whereas controls only performed a single trial in each experimental condition. To minimize patient fatigue, a single trial in each condition was carried out. We followed a previous study that adopted single trials to examine the effect of mirrored visual feedback on phantom limb sensations (Hunter et al., 2003).

During the experimental conditions, participants viewed three types of video images of the participant’s own hand movement: 0 ms (synchronous), 250-ms delayed, and 500-ms delayed images. In the 250-ms and 500-ms delayed conditions, the patient viewed the image with a mental set in which he adjusted the phase of the phantom limb clasping and unclasping to that of the delayed image (adjusted action) and viewed the image without this mental set (unadjusted action). For example, in the 500-ms delay condition with adjusted action, the patient clasped and unclasped alternately the left phantom and right intact limbs. During unadjusted action, the patient also clasped and unclasped the left phantom and right intact limbs at the same rate as the right hand movement. On the other hand, controls adopted a mental set in which they adjusted the phase of motor imagery of the left arm clasping and unclasping to that of the delayed image during adjusted action.

During the control conditions, the patient performed the same clasp/unclasping task in two ways. One was a non-video condition in which a blank monitor with a black screen was presented. The other was a real mirror condition, similar to traditional mirror therapy, in which a transportable mirror (25 × 40 cm) was placed in the patient’s sagittal plane against the standing screen.

### Subjective ratings of phantom motor sensation

The patient subjectively rated phantom motor sensations after each trial. After the patient stopped clasping and unclasping, the intensity of felt motor sensation in the phantom limb using magnitude estimation was rated. The patient received the following instructions: “How strongly did you feel like controlling your phantom limb during the task?” Prior to these trials, the patient experienced phantom motor sensation in the synchronous condition without a delay. Next, the patient performed a trial in one of the experimental and control conditions, and then rated the intensity of the phantom motor sensation in the condition relative to the synchronous condition, which was supposed to have a rating of 10. To rate the subjective intensity of phantom motor sensation, the patient was allowed to use arbitrary scores above 0. Higher scores indicated stronger intensity of phantom motor sensation, and 0 indicated no sensation at all.

### Electromyographic recording

To examine the relationship between phantom motor sensation and actual muscle activity, we recorded muscle activity in the left anterior and posterior deltoid of both the patient and control group during the trials. Because the patient had a limb amputation below the left shoulder, and showed obvious movement of the deltoid during motor execution of the phantom limb during mirror therapy, we chose the deltoid for EMG recordings.

To compare the EMG waveform of the left deltoid with the phase of alternation in the right hand clasping and unclasping, we also recorded EMG of the right flexor digitorum superficialis (FDS) and extensor digitorum communis (EDC) in both the patient and control group after completion of all trials. During this recording, participants kept clasping and unclasping their right hand for 10 s along with the metronome but without a video image.

EMG signals were captured with disposable Ag/AgCl surface electrodes (P-150, Nihon Koden Corporation) placed in a bipolar configuration. EMG data were recorded using a data acquisition system (MP150, BIOPAC Systems Inc.) and an electromyogram amplifier (EMG2, BIOPAC Systems Inc.). EMG signals were recorded at a frequency of 1000 Hz and band pass filtered between 20 and 400 Hz.

### Data analysis

Subjective ratings regarding the intensity of phantom motor sensation and EMG recordings for single trials in each experimental and control conditions were obtained from the patient. The patient’s ratings were reported descriptively and compared across conditions without statistics. To corroborate ratings varying across conditions by consistent EMG variations, we used a Pearson’s product-moment correlation coefficient to determine the relationship between the ratings of phantom motor sensation and EMG indices from the patient’s stump muscles, using maximum peak amplitude (MAX) and root mean square amplitude (RMS) during each trial. Finally, we confirmed that EMG activity from control participants’ deltoids was not observed across the experimental conditions by visual inspection.

## Results

### Subjective ratings of phantom motor sensation

**Figure 2:**
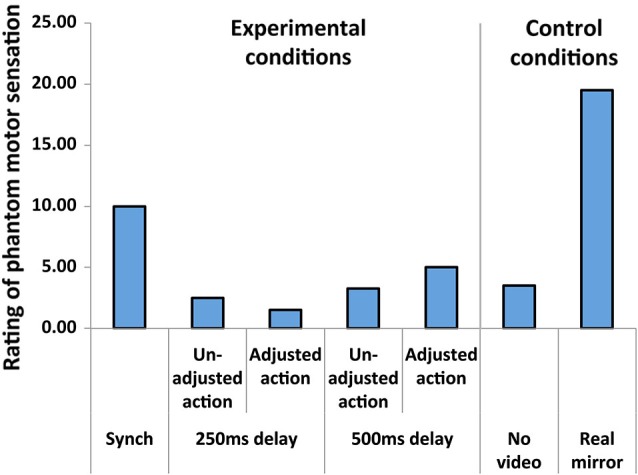
**The patient’s subjective ratings of intensity in phantom motor sensation as a function of condition**.

Figure 2 presents the patient’s results from single trials in the experimental and control conditions. In the 250-ms and 500-ms delayed conditions, sensations decreased to 2.50 and 3.25, respectively, relative to the standard in the synchronous condition (10.00). The patient reported an odd feeling regarding the delayed appearance of the hand images during debriefing. However, when the patient performed action in which he adjusted the phantom movement to visual feedback with a 500-ms delay, the rating increased to 5.00. This effect due to the adjusted action was not observed in the 250-ms delayed condition (the rating slightly decreased to 1.50). When the patient performed the task while viewing his hand in the real mirror, the rating increased to 19.50. The patient was comfortable during the real-mirror condition and reported vivid phantom motor sensation during debriefing. In the non-video condition in which the monitor presented only a black screen, the rating was 3.50.

### Electromyographic recording

**Figure 3:**
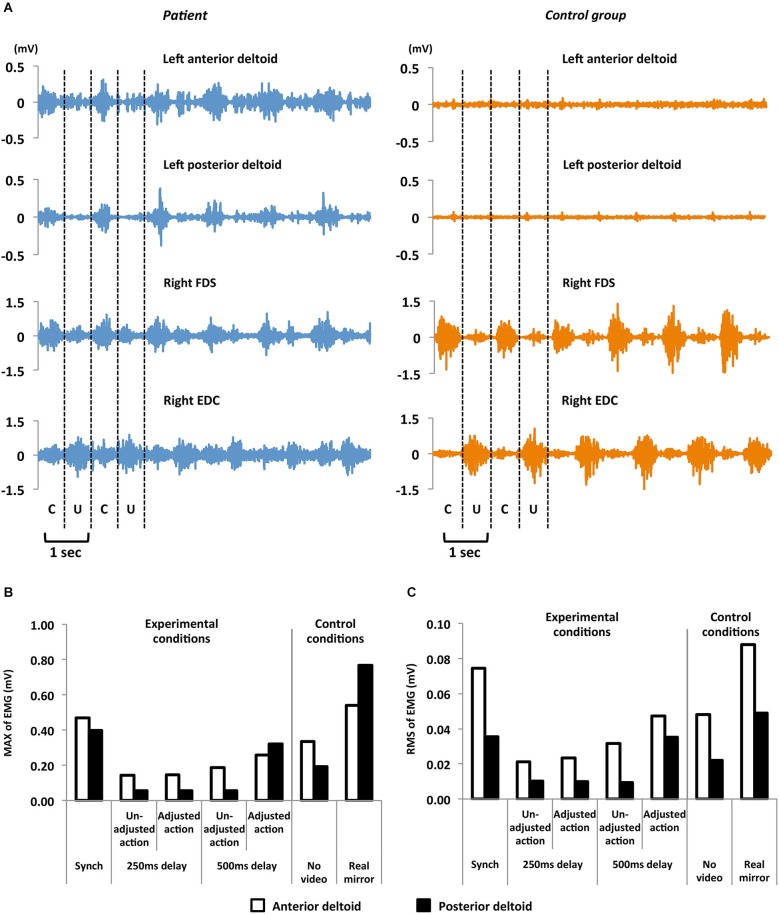
**(A)** EMG activity in the left anterior and posterior deltoids, and right FDS and EDC in the patient (left column) and one control participant (right column). EMG waveforms of the left deltoid were obtained when participants performed the task with synchronous visual feedback. The sinusoidal EMG waveforms correspond to phase clasping (C) and unclasping (U). **(B)** MAX of EMG activity recorded at the patient’s anterior and posterior deltoid as a function of condition. **(C)** RMS of EMG activity recorded at the patient’s anterior and posterior deltoid as a function of condition. The ratings significantly and positively correlated with each MAX and RMS at both the anterior and posterior deltoid (*r* ≥ 0.87, *p* < 0.01).

The EMG recorded on the patient’s left anterior and posterior deltoid showed greater activity during each condition than at rest. Figure 3A shows a waveform of EMG activity in the left deltoid and right FDS and EDC in the patient and one control participant. Overall, EMG at the patient’s left deltoid showed sinusoidal waveforms corresponding to phase alternation of clasping and unclasping. Thus, we attributed this muscle activity to phantom motor sensations.

Figures 3B,C present the patient’s EMG activity results from single trials in both experimental and control conditions. EMG activity was greater when subjective sensation ratings were higher: there was high correspondence between phantom motor sensation and EMG activity. We also analyzed the relationships between subjective ratings of phantom motor sensation and the MAX and RMS of EMG during each trial. We found significant and strong positive correlations between subjective ratings and MAX at the anterior deltoid (Pearson’s coefficient *r* = 0.90, *p* < 0.01), MAX at the posterior deltoid (*r* = 0.97, *p* < 0.01), RMS at the anterior deltoid (*r* = 0.92, *p* < 0.01), and RMS at the posterior deltoid (*r* = 0.87, *p* < 0.01).

In the control group, no EMG activity was observed at both the anterior and posterior left deltoid during all conditions (Figure 3A). For further demonstration of a lack of EMG activity among control participants, we conducted two additional tasks. First, we removed the monitor and then asked control participants to repetitively clasp and unclasp their left hand with the left elbow placed on the table during EMG recording at the left deltoid. No EMG activity was observed at the left deltoid. Second, we recorded EMG activity at the left FDS and EDC while performing the same task in the same experimental conditions. Again, no EMG activity was observed in the FDS and EDC. These results confirmed that EMG activity was uniquely evoked in the patient’s left deltoid and was related to phantom limb sensations.

## Discussion

Using a video-projection system, the present case study investigated the effect of visual feedback on perceived phantom motor sensation (i.e., a sense of controlling a phantom limb). Our results suggest three findings. First, delayed visual feedback reduced phantom motor sensation in our patient. The patient perceived decreased phantom motor sensation when visual feedback was delayed by 250 and 500 ms. These results are consistent with previous findings suggesting that delayed sensory feedback of an action decreases a sense of agency (Franck et al., 2001; Sato and Yasuda, 2005; Asai and Tanno, 2007). Since temporal contiguity between an action and sensory feedback predicted by the internal forward model is necessary for generating a sense of agency (Wolpert et al., 1995; Wolpert, 1997; Frith et al., 2000), action through a phantom limb should also predict sensory feedback if temporally matched. Several previous studies investigated the relationship between visual feedback of an action and phantom motor sensation. For instance, Kawashima et al. (2013) reported that amputees perceived stronger phantom motor sensation with mirrored visual feedback than without such feedback. However, to our knowledge, no study has examined the relationship between the temporal properties of visual feedback for phantom limb movement and phantom motor sensation. For the first time, we show that phantom motor sensation requires temporal contiguity between the motor intention and visual feedback.

Second, we found that even if delayed visual feedback reduced phantom motor sensation, the patient’s spontaneous effort to adjust the phase of phantom movement to that of delayed feedback (adjusted action) helped restore phantom motor sensations. This result was consistent with previous findings suggesting that a sense of effort enhances a sense of agency among healthy individuals (Demanet et al., 2013; Damen et al., 2014). However, the restoration was observed only during a 500-ms delay. We assumed that difficulty in spontaneously adjusting phantom limb movement to delayed visual feedback would affect phantom motor sensation. That is, for the hand image paired with a 500-ms delay, the actual hand appeared to be alternately clasping and unclasping at a tempo of 120 beats per minute. However, the 250-ms delayed image presented a lag with an alternation of clasping and unclasping with two hands. In this sense, a larger delay seems advantageous for generating a sense of agency. However, more delayed visual feedback tends to increase the discrepancy between the action and sensory feedback, subsequently inducing a decreased sense of agency (Sato and Yasuda, 2005; Asai and Tanno, 2007). Thus, our results may reflect the influence of top-down processing (Synofzik et al., 2008) in which the patient intended to judge the 500-ms delayed image as self-produced movement. A recent study also reported that delayed visual feedback decreased manual task performance (Fujisaki, 2012). Performance sharply decreased with increasing delay up until 490 ms and decreased more gradually as the delay increased up to 2120 ms. In a sense, this previous finding is consistent with ours in that the adjusted action to a delayed hand image can only be influential during a certain time window (500 ms); however, there are apparent differences in the time window and phantom/intact limbs used in Fujisaki (2012) and the present study. Our results suggest that the patient’s mental set toward mirrored visual feedback might help elicit phantom motor sensation, which is necessary for therapeutic benefits (e.g., alleviate phantom limb pain) during both mirror (Ramachandran and Rogers-Ramachandran, 1996) and video-projection system therapy (Giraux and Sirigu, 2003; Mercier and Sirigu, 2009), as well as virtual reality (Murray et al., 2007; Cole et al., 2009).

Finally, we observed that EMG activity in the patient’s stump muscles correlated with the phase of phantom limb clasp/unclasping movements, consistent with previous findings that upper-limb stump EMGs correlate with the phantom limb movement phase (Kawashima and Mita, 2009; Raffin et al., 2012a; Kawashima et al., 2013). More importantly, our results showed that EMGs were highly correlated with modulations in both phantom motor sensation through visual feedback and the intention to adjust phantom movement to delayed visual feedback. A recent study also reported that ease of phantom limb motor execution is positively correlated with EMG activity in a forearm stump when compared with and without mirror visual feedback (Kawashima et al., 2013). Furthermore, we confirmed that this modulated EMG activity in the deltoid was not observed among healthy controls. Consistent with previous findings (Gagné et al., 2009), these results suggest that phantom motor sensation is involved in certain motor commands targeted at stump muscles originally not targeted prior to amputation. These motor commands are capable of not only decreasing when perceived phantom motor sensation attenuates, but also increasing when the individual intends to facilitate phantom limb movement.

Our study has three limitations of note. First, in our single-case study, one patient performed one trial in each condition. Consequently, while a significant correlation between subjective ratings of phantom motor sensation and EMG activity on the stump was demonstrated, we were unable to determine statistically significant differences among conditions. Therefore, further research with a larger sample size is necessary to examine the reliability of our results. Second, because the patient was not experiencing phantom limb pain at the time of the study, we could not investigate the therapeutic effect of the video-monitor technique. Further investigations should be conducted to identify how phantom motor sensations remedy phantom limb pain, comparing the efficacy of a video-projection system and mirror therapy. Since mirror therapy requires a sensorimotor coherence (Ramachandran and Altschuler, 2009), phantom motor sensation in terms of producing a sense of agency should be important in the treatment of phantom limb pain. Finally, we did not examine whether a sense of body-ownership toward the hand images could have emerged in the patient. This was because we measured only phantom motor sensation as one of the components related to a sense of agency. Given that coherent body-ownership is based on a sense of agency (Tsakiris et al., 2006), future studies should investigate the relationship between phantom motor sensations and a sense of ownership toward an external alternative to a phantom limb. Additional studies should also examine how a sense of agency and body-ownership contribute to mirror therapy effects.

Given that the strongest sensations were observed for the real mirror condition, the video-projection system cannot easily replace traditional mirror therapy. For instance, our system could not precisely reproduce the appearance of an object (e.g., spatial resolution and three-dimensional effects). Since it has been suggested that susceptibility to visual feedback might relate to the effectiveness of virtual visual feedback for therapeutic use (Mercier and Sirigu, 2009), video-projection visual feedback must be able to reproduce more vivid and exact feedback. However, the video-projection system, which can manipulate the timing of visual feedback unlike a real mirror, might be beneficial for not only the investigation of relationships between a phantom limb and a sense of agency but also for the development of new therapeutic approaches that use top-down processing, such as a sense of effort (Demanet et al., 2013; Damen et al., 2014) and contextual prediction (Blakemore et al., 1998). Moreover, this system, which can present pre-recorded visual feedback of an action, might be beneficial for bilateral amputees and patients with bilateral motor impairment.

In conclusion, the present single-case study suggests that a sense of agency over a phantom limb and EMG activity on stump muscles depends on visuomotor synchrony and a mental set for adjusting phantom limb movement to delayed visual feedback. This indicates that a sense of controlling an external proxy (e.g., hand images) can be generated even though the agent does not have an effector (e.g., intact limb). Several findings regarding a sense of agency can be applied to investigate mechanisms related to a phantom limb, providing therapeutic applications based on future studies.

## Conflict of interest statement

The authors declare that the research was conducted in the absence of any commercial or financial relationships that could be construed as a potential conflict of interest.
